# High-resolution seismic event detection using local similarity for Large-N arrays

**DOI:** 10.1038/s41598-018-19728-w

**Published:** 2018-01-26

**Authors:** Zefeng Li, Zhigang Peng, Dan Hollis, Lijun Zhu, James McClellan

**Affiliations:** 1 School of Earth and Atmospheric Sciences, Georgia Institute of Technology, Atlanta, GA USA; 2 Seismological Laboratory, California Institute of Technology, Pasadena, CA USA; 3 Sisprobe SAS, San Gabriel, CA USA; 4 Center for Energy and Geo Processing at Georgia Tech and King Fahd University of Petroleum and Minerals, Atlanta, GA USA

## Abstract

We develop a novel method for seismic event detection that can be applied to large-N arrays. The method is based on a new detection function named local similarity, which quantifies the signal consistency between the examined station and its nearest neighbors. Using the 5200-station Long Beach nodal array, we demonstrate that stacked local similarity functions can be used to detect seismic events with amplitudes near or below noise levels. We apply the method to one-week continuous data around the 03/11/2011 Mw 9.1 Tohoku-Oki earthquake, to detect local and distant events. In the 5–10 Hz range, we detect various events of natural and anthropogenic origins, but without a clear increase in local seismicity during and following the surface waves of the Tohoku-Oki mainshock. In the 1-Hz low-pass-filtered range, we detect numerous events, likely representing aftershocks from the Tohoku-Oki mainshock region. This high-resolution detection technique can be applied to both ultra-dense and regular array recordings for monitoring ultra-weak micro-seismicity and detecting unusual seismic events in noisy environments.

## Introduction

Seismic arrays have provided important data for studying the Earth’s structures and earthquake processes since the 1960s^1^. Based on recordings from closely spaced uniform seismometers, various array methods can effectively enhance signal-to-noise ratios and significantly lower the detection threshold. Dense regional networks such as the Japanese Hi-net array and the Southern California Seismic Network, as well as the recent US-Array, have been increasingly used to monitor regional seismicity in real time and provide high-quality earthquake catalogs. Dense arrays are also useful for detecting new types of seismic events such as deep tectonic tremor^2^. With advances in instrumentation technology, the density of seismometers in a given array has increased dramatically in the past decades. In recent years, ultra-dense arrays with hundreds to thousands of sensors and tens- to hundred-meter interstation spacing have been deployed in several regions. One of the notable examples is the Long Beach 3D array, which contains 5200 sensors with 100 m spacing covering the urban area of the City of Long Beach^3^. Such arrays, sometimes called large-N arrays, provide unprecedented detection capability for small magnitude earthquakes and other unconventional sources^4–6^.

Apart from instrumentation technology, data processing methods also influence the resolution of seismic event detection. Traditional earthquake detection workflow includes phase picking (identifying impulsive arrivals of seismic phases) and phase association (grouping these phases into an individual event)^7^. Phase picking is commonly done by an energy detector such as the short-term average over long-term average (STA/LTA) ratio^8,9^. As computer power increases, many sophisticated detection algorithms have been proposed. Most of these algorithms continuously search over possible source locations by shifting and stacking waveforms or their variants (e.g., envelope, STA/LTA, normalized waveform). Outstanding examples include the source scanning algorithm^10^, coalescence microseismic mapping^11^, and backprojection^12,13^.

Different from this category, template matching, or matched filtering, takes advantage of predetermined events and cross-correlates them with continuous recordings to detect events with high waveform similarities^14^. This is based on the fact that nearby seismic events should have similar source mechanisms and ray paths, and hence similar waveforms. Template matching techniques have been widely applied to detect emergent tremor and small earthquakes, and usually result in new events at a factor of 5–10 times the original catalog^15–17^. However, template matching requires predetermined templates as inputs, which are not always available. In addition, it tends to detect events that are similar to templates, which may bias the detection results. An auto-detection technique could be used to build template events from scratch^18^. However, with *O*(*N*^2^) type scaling, this is computationally very intensive and hence cannot be applied to longer time series^19^. Other approaches, such as fingerprinting^19^ or earthquake search-engines^20^ have also been proposed for fast and robust detection of seismic events.

The emergence of large-N arrays (i.e., a large number of sensors and short station spacing) provides unprecedented opportunities for high-resolution structural imaging and microearthquake detection. Meanwhile, it also poses new challenges for processing methods. Recent studies attempted to use advanced signal processing techniques, such as subarray analysis and graph clustering, to detect and locate sources within dense large-N arrays^21,22^. Here we introduce a new method that takes advantage of the primary features of emerging large-N arrays. The method involves a new metric termed local similarity. It evaluates the similarity on a given station with respect to its nearest neighbors. This is different from conventional metrics that consider each station individually, or standard array processing methods that consider all stations together with some general assumptions^1^ (e.g., plane waves or spherical wavefront with predicted arrivals).

**Figure 1:**
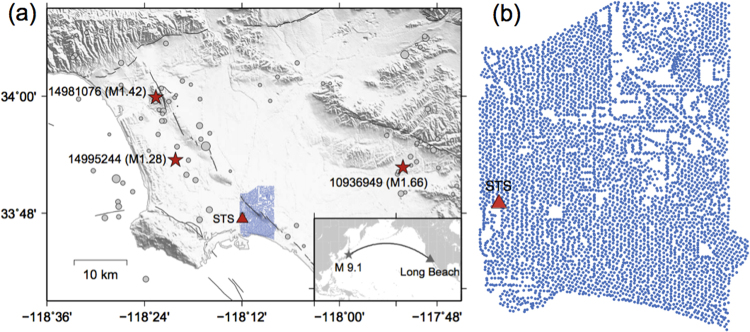
Map of study region, the Long Beach nodal array and local seismicity. (**a**) Blue dots are the 5200-sensor nodal array. A red triangle mark the broadband station STS belonging to Southern California Seismic Network (SCSN). Black curves denote the surface trace of mapped faults. Gray solid circles are seismicity listed in the SCSN catalog between January and June 2011, whose sizes are proportional to magnitude. Red stars mark three selected cataloged events used for tests. The inset map shows locations of the M 9.1 Tohoku-Oki earthquake and its ray path to Long Beach. (**b**) A zoom-in plot of the Long Beach array and the STS station. Figure was made using the Generic Mapping Tools version 4.2.1^33^ (http://gmt.soest.hawaii.edu/).

We apply this method to one-week continuous data between 03/06/2011 and 03/12/2011 recorded by the 5200-station Long Beach array^3^. The Long Beach array was deployed from January to June 2011 as part of a petroleum exploration survey, which contained 5200 geophones with a 10-Hz corner frequency (Fig. 1). This array covers 7 × 10 km in the city of Long Beach, with a nominal interstation distance of 100 m. Several segments of the Newport-Inglewood fault pass through this area. The 1933 Mw 6.4 Long Beach earthquake occurred about 10 km to the southeast of the array. Because the area is densely populated and the sensors were simply buried very close to the surface, the recordings are heavily contaminated by large-amplitude anthropogenic noise, which poses a challenge for seismic detection^4^.

We choose the one-week time period in March mainly because of the occurrence of the Mw 9.1 Tohoku-Oki earthquake on 03/11/2011. This event has triggered numerous microearthquakes and deep tectonic tremor around the world^23^, including the San Jacinto Fault in southern California^24^. Systematical examination of remote triggering potential in California found that in addition to geothermal/volcanic regions, the Los Angeles basin has an unusually high triggering susceptibility compared to surrounding regions^25^. Hence, it is reasonable to assume that the Tohoku-Oki mainshock could have triggered some microseismicity in this region, although these events may not be detected with conventional methods/arrays.

In the following sections, we first show that detecting events below the noise level is feasible via direct stacking of local similarity. Then we perform a systematic comparison with template matching and STA/LTA. Finally, we apply it to one week of continuous data of the Long Beach array to detect local and distant events, and examine whether the Tohoku-Oki mainshock may have triggered any significant increase of microseismicity in this region.

## Methods

**Figure 2:**
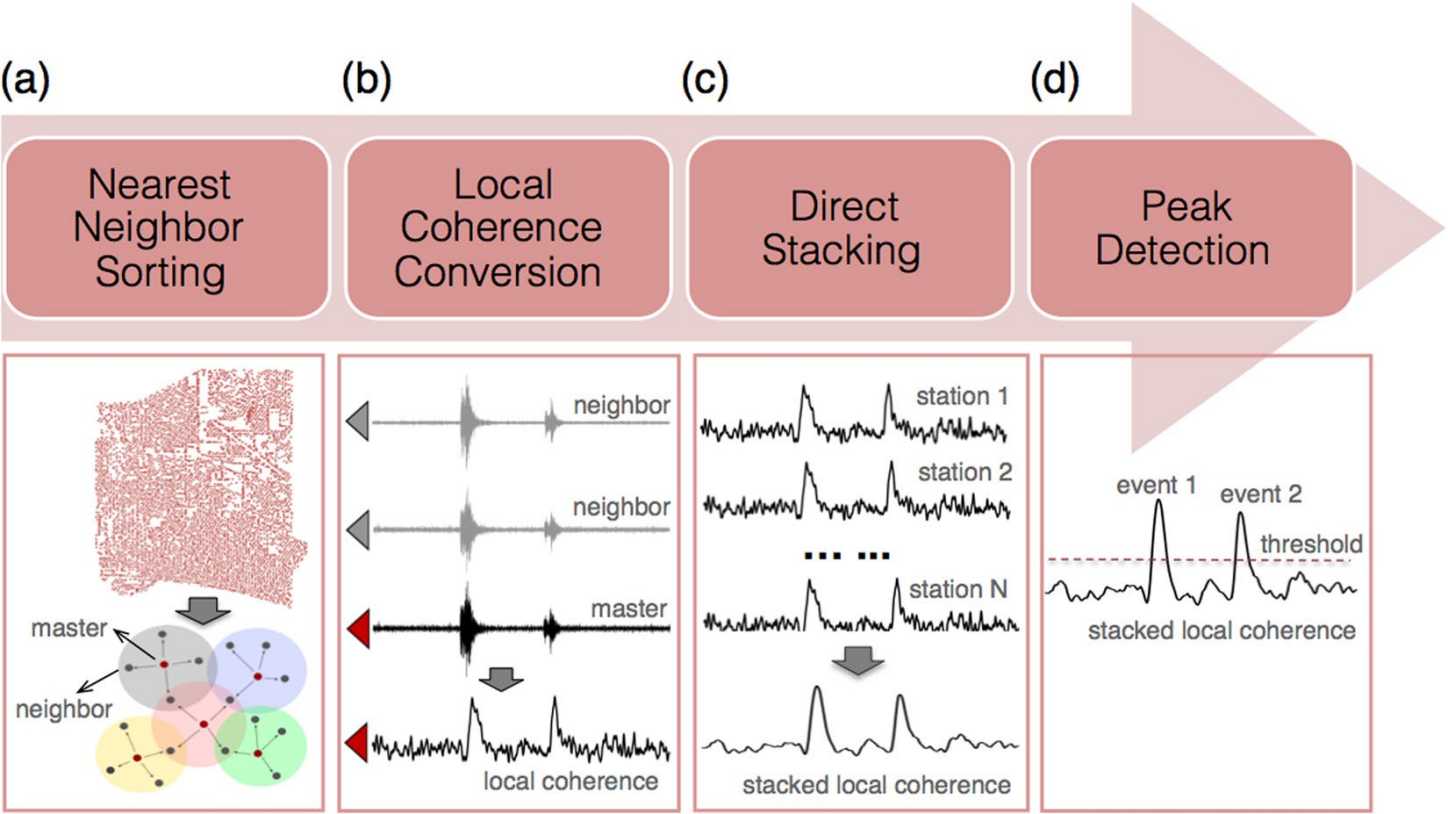
Flowchart and schematic diagrams of local similarity detection. (**a**) For each master station, find its nearest neighbors for local similarity computation. (**b**) Convert the seismic waveforms on the master station into local similarity by cross-correlation and averaging of those of its neighbors. (**c**) Stack local similarity of all stations. (**d**) Apply sliding-window threshold to select positive detections.

For a pair of two spatially close stations, the ray paths for a common source are very similar. Hence, their waveforms are expected to be nearly identical, while random noise sources at these sites remain sufficiently different. Hence in principle, we can distinguish a signal from noise by measuring the waveform similarity on neighboring stations, which is termed local similarity (Fig. 2a and b). Note that here we do not require waveform similarity across the entire network^1,26^. Mathematically, local similarity at a master station is defined as the average of sliding-window normalized cross-correlations (CCs) with its nearest neighbors. Each sliding time window is allowed to shift within a time lag relative to its neighboring recordings, in order to account for arrival time differences between neighboring stations. The peak cross-correlation function between a master station and a neighboring station is defined as

1 $${s}_{ij}(t)=\mathop{\max }\limits_{-L\le l\le L}\frac{|{\sum }_{m=-M}^{M}{u}_{i}(t+m\delta ){u}_{ij}(t+m\delta +l\delta )|}{\sqrt{{\sum }_{m=-M}^{M}{u}_{i}^{2}(t+m\delta ){\sum }_{m=-M}^{M}{u}_{ij}^{2}(t+m\delta +l\delta )}}$$

where *u*_*i*_ is the recording of the *i*^*th*^ master station, and *u*_*ij*_ is the recording of the *j*^*th*^ neighbor of the *i*^*th*^ master station, *δ* is the sampling interval, (2*M* + 1)*δ* is the sliding window length, and *Lδ* is the maximal time lag, which is determined by the upper limit of wave slowness and distance from the *j*^*th*^ neighboring station to the master station *i*. In this study, we set the sliding window length as 1 s and 3 s for the high-frequency (5–10 Hz) and long-period (<1 Hz) signal, respectively. The peak correlation value within the time range is taken as the value for that sliding window. The resulting correlations between the master and its neighboring stations are averaged into a single value, termed the local similarity *S*_*i*_ (Equation 2):

2 $${S}_{i}(t)=\frac{1}{K}\sum _{j=1}^{K}{s}_{ij}(t),\,i=1,2,\,\ldots ,\,N;j=1,\,2,\,\ldots ,\,K$$

where *K* is the number of nearest neighbors, which is set by the user. Typically, we use four neighbors for a 2D array and two neighbors for a 1D array (Fig. 2a). Eight neighbors are also used in 2D arrays^22^, but adding neighbors that are further away may violate the waveform similarity assumption in our method.

The resulting average CC trace represents the signal resemblance of the master station with respect to its neighbors in continuous time. After obtaining local similarity traces for all the stations, we directly stack them without any shifts to obtain a representative network trace (Fig. 2c). Direct stacking enables us to perform a general-purpose detection without assuming any wave types and velocity models in the study region.

Event detection in template matching is done by applying a common threshold to the network-stacked cross-correlation trace, which is generally defined as the median plus several times the median absolute deviation (MAD). Such thresholding is not suitable for local similarity because local similarity tends to have a fluctuating trend due to temporal drift in the similarity of background noise. To remove this long period trend, we first perform a weighted least-squares fit of the trace with a tenth-order polynomial, and then subtract the fitting curve from the trace. The tenth-order polynomial applied to the hourly data primarily fits components with periods longer than 18 mins (Figure S4). Tenth-order is an empirical choice and small changes in the order have very little influence on the results. After that, we apply a 1-minute sliding time window to select the outstanding peaks. For every window, the threshold is defined by the MAD and median calculated for that window. Statistically, a higher significance level suggests more stable detection and potentially fewer false alarms. We use the median plus ten times the MAD as the threshold for event detection in this study (Fig. 2d).

## Results

### Comparisons with STA/LTA, envelope function and template matching

**Figure 3:**
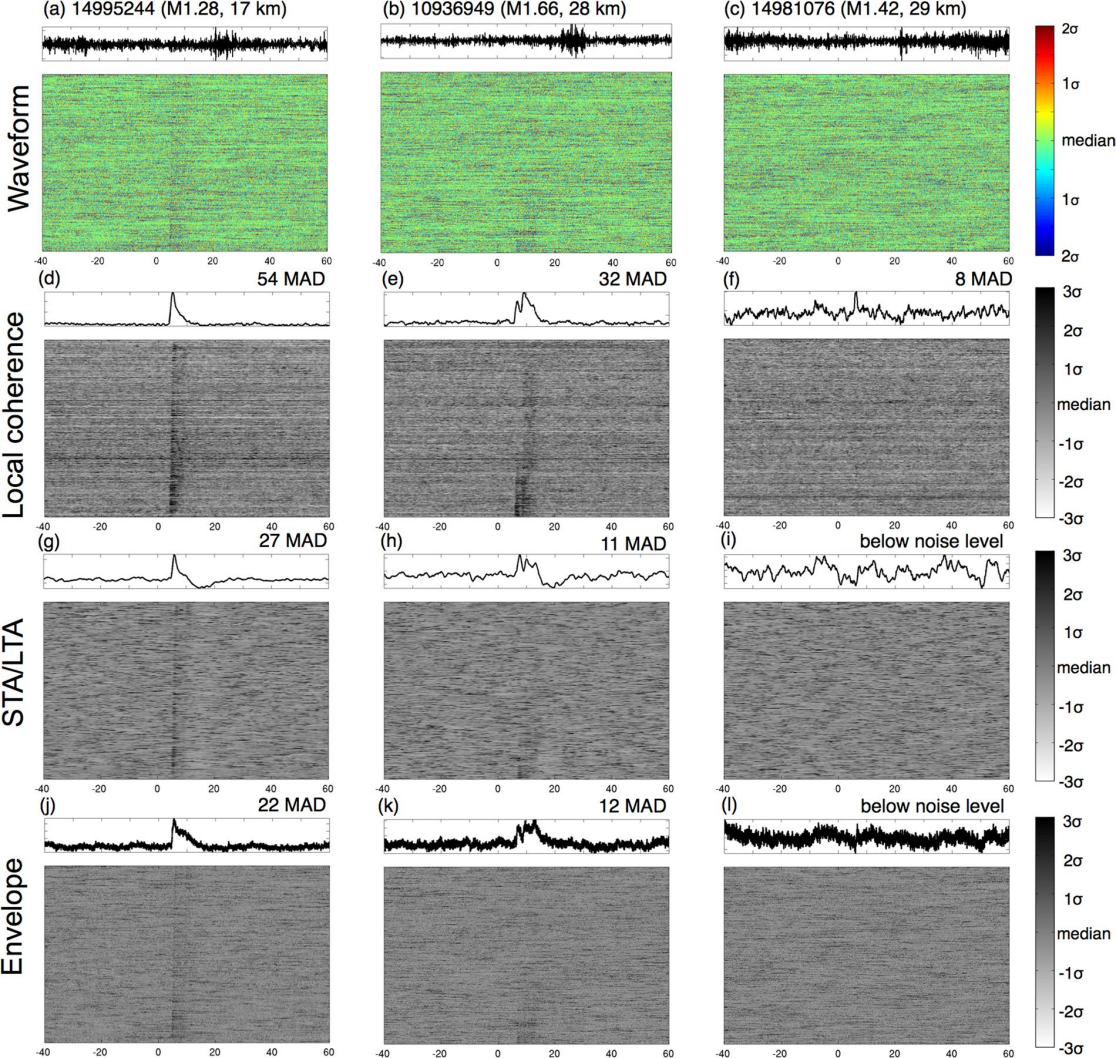
Performance tests of local similarity, short-term-average/long-term-average (STA/LTA), and envelope for three catalogued weak events. Traces along Y-axis are sorted by their epicentral distances. (**a**–**c**): raw waveforms of the events plotted in color for the Long Beach array. The upper curves in the box are raw recordings at station STS. Magnitude and distance from the array center are listed beside the event IDs. The waveforms are plotted according to their normalized amplitudes relative to the median. (**d**–**g**): local similarity corresponding the waveforms in (**a**–**c**). The black curves at the top are the stacked local similarity of the whole network. The significance levels of the peaks are marked. (**g**–**i**) and (**j**–**l**): for STA/LTA and envelope, respectively.

We use three local events listed in the SCSN catalog (SCEC ID: 14995244, 10936949 and 14981076) to evaluate the performance of local similarity. We select these events as they are 20–50 km from the network (Fig. 1) and have magnitude less than 2, so the waveforms recorded by the Long Beach array have very weak amplitudes. Figure 3a–c shows that the recordings are heavily contaminated by incoherent noise. We then examine how these events are manifested by local similarity, STA/LTA and envelope functions, respectively. For each method, we first convert the waveforms of individual stations into a metric function and stack them into a network representative trace. For STA/LTA, we use 10 s for LTA and 1 s for STA. On the stacked trace, we measure the peak significance of the target event relative to the background level by evaluating (peak-median)/MAD, which is the number of MADs above the median.

Figure 3 shows that for the three target events, the results from STA/LTA are comparable to those from the envelope function. They both have reasonable peaks for events 14995244, 10936949, but do not show any discernable patterns for event 14981076. In comparison, local similarity shows significant peaks for all three events and has peak significance twice or more than that of STA/LTA and envelope. Thus local similarity produces more reliable detection than the other two metrics.

To further quantify the performance, we carry out synthetic tests with different signal-to-noise ratios (SNRs) for local similarity, STA/LTA, and template matching. Here we remove the envelope due to its comparable performance with STA/LTA and include template matching in the comparison. We note that because template matching uses prior information, i.e., known target waveforms, it should be treated as an optimal benchmark for the other two methods.

**Figure 4:**
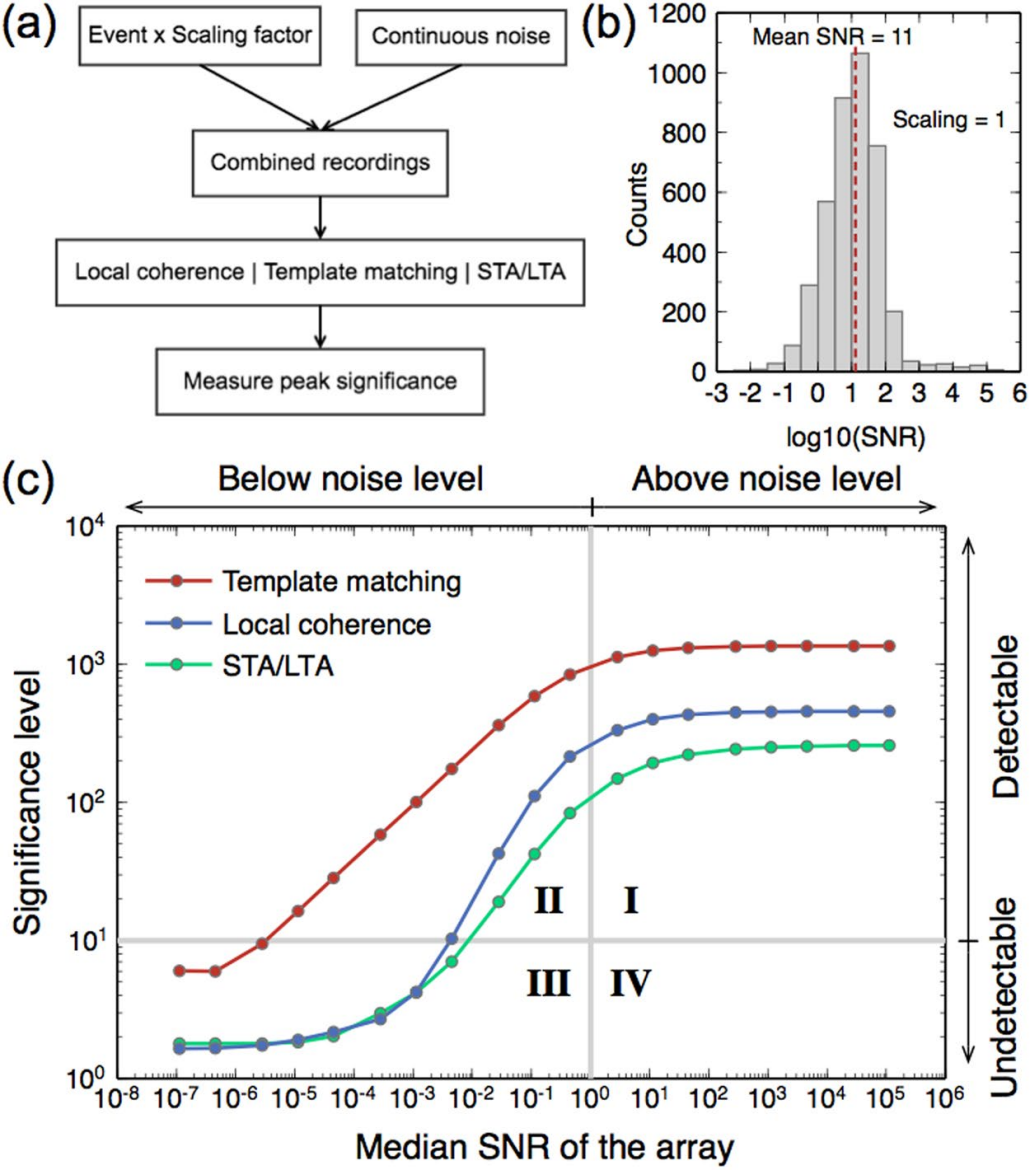
Synthetic test of detection performance of local similarity, template matching, and STA/LTA as a function of the median signal-to-noise ratio (SNR) of the array. (**a**) Flowchart of the synthetic test. Note event waveform is scaled by different factors to create recordings with different array median SNRs. (**b**) Histogram of SNR for 4000 stations when the scaling factor is 1. (**c**) Significance level versus SNR from local similarity, template matching, and STA/LTA. The vertical and horizontal gray lines mark SNR = 1, and detection threshold = 10 MAD, respectively.

We generate synthetic data according to the following steps^27^: (1) select a local event with good SNR (ID 14930284, M 2.24), and multiply the waveforms by a scaling factor; (2) add the waveform on top of a randomly selected continuous noise at each station; (3) apply local similarity, STA/LTA and template matching method to the data obtained in step (2), resulting in a stacked trace; (4) measure the peak significance on the stacked trace at the time when the event is added. This process is outlined in Fig. 4a. Note that the scaling factor is changed for every test to generate data with different SNRs. When the scaling factor is 1, the SNR distribution of 4053 stations has a median SNR at 11, with a dominant range from 1 to 100 (Fig. 4b). Different scaling factors just shift the distributions of SNR. Hence, the peak significance for each method can be characterized as a function of the median SNR of the array (Fig. 4c). As expected, template matching outperforms the other two methods. In this particular case, template matching has a detection capability down to SNR~10^-5^ if the threshold of positive detection is set at 10 MAD above the median. Local similarity, without the requirement of any prior knowledge, can detect events down to SNR~0.01. It ranks the second and generally has a detection significance level more than twice that of STA/LTA (except at very low SNRs), which is consistent with the tests using three catalog events (Fig. 3). These results show that our local similarity method can detect events far below noise level without prior information or assumption, which is different from traditional array-based methods or recent template-matching techniques. Therefore, we name it as “high-resolution detection”.

**Figure 5:**
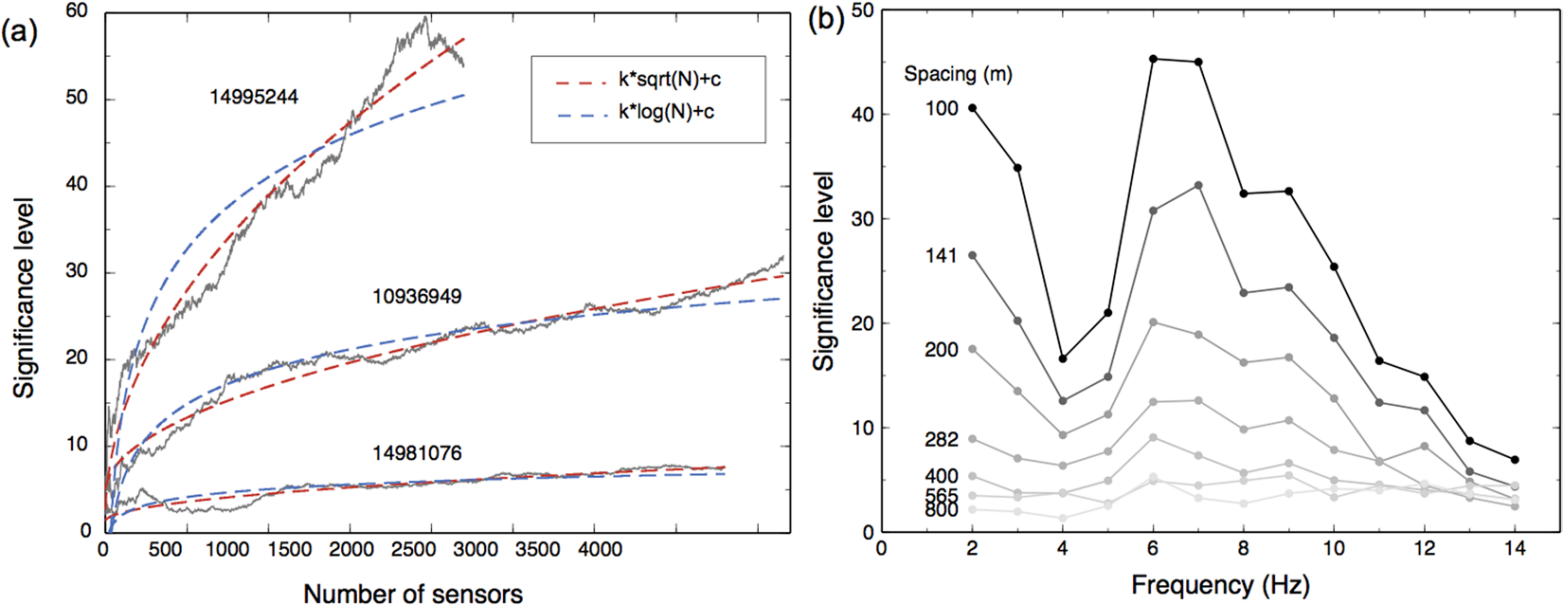
Performance tests of local similarity dependence on array settings and waveform frequencies. (**a**) Tests of significance level dependence on the number of sensors while maintaining the same station spacing. The red and blue dashed lines are sqrt(N)- and log(N)- type fitting curves, where N is the number of sensors. We note that sqrt(N) generally performs better than log(N) does. (**b**) Tests of significance level dependence on the station spacing (while maintaining the same array aperture) and frequency bands.

### Performance dependence on the number of sensors, sensor spacing, and frequencies

We perform additional tests to examine the method’s performance depending on array settings and waveform frequencies. To evaluate the dependence on the number of array elements, we change the number of array sensors by removing sensors at the peripherals and measure the significance level of the events on the summed similarity trace. We perform the test on three example events shown in Fig. 3 and obtain similar trends. Figure 5a shows that, with an increase in the number of sensors, the significance of the detection also increases, as expected. We also fit the results with sqrt(N) and log(N) functions, and find sqrt(N) generally performs better job than log(N), where N is the number of sensors. This suggests that, with increasing number of sensors, the incremental performance gain per added sensor decreases gradually.

To test the dependence on station spacing and frequency bands, we down sample the sensors spatially (while maintaining the same array aperture), and filter the data into multiple narrow frequency bands. The original number of array sensors is ~4200 (spacing is ~100 m). We down sample the sensors by a factors of 2, 4, 8, …, 64, resulting in spacings 141, 200, 282, …, 800 m. The frequency bands are chosen as 1–3, 2–4, 3–5, …, 13–15 Hz. For each dataset, we perform the detection test and measure the significance of peaks. The results summarized in Fig. 5b show that with decreasing spacing, performance generally decreases, as expected. The performance also depends on the examined frequencies band, because the SNR varies for different frequencies.

### Detection of local and distant seismicity

**Figure 6:**
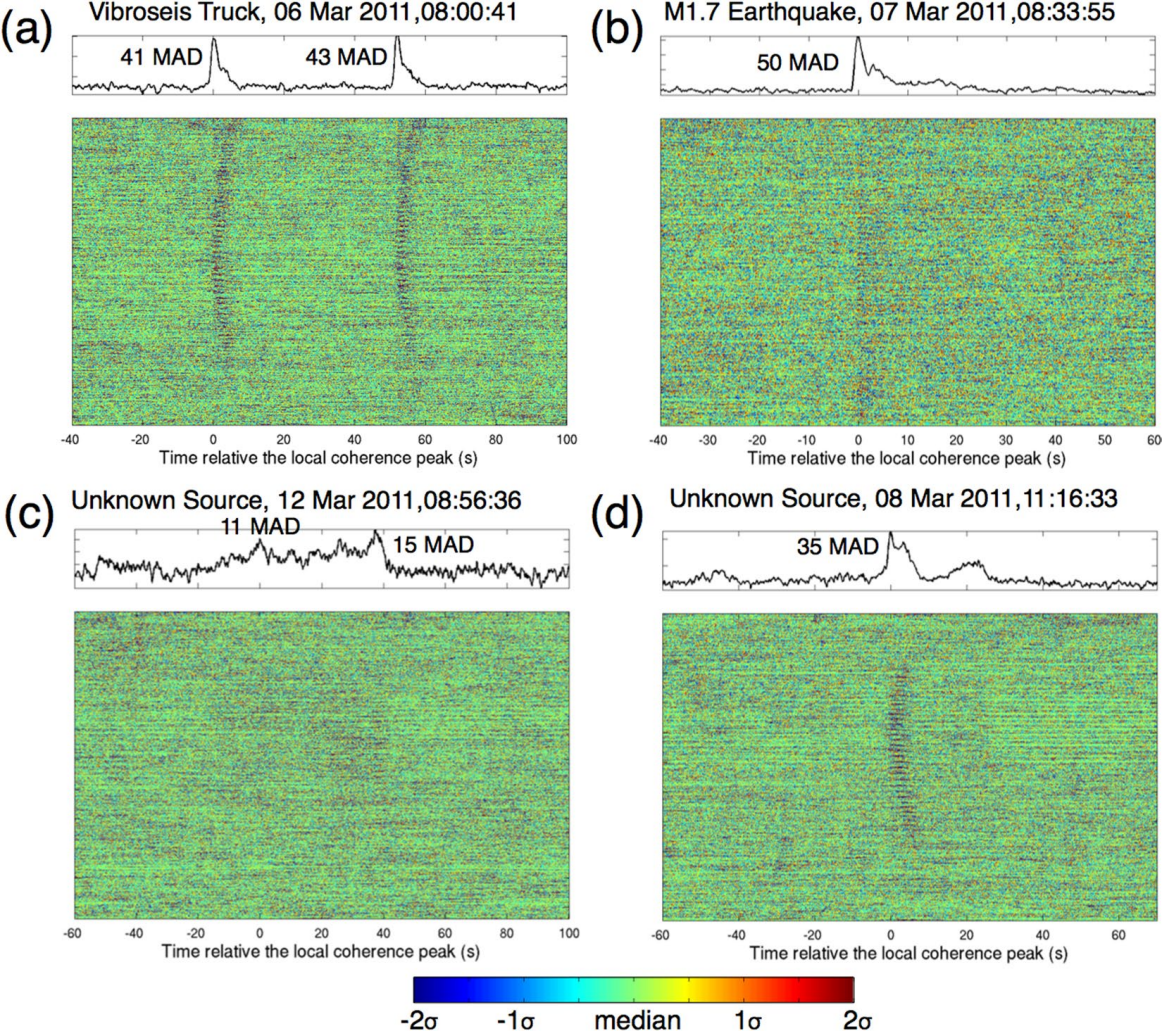
Detected examples of local events in 5–10 Hz filtered data. (**a**) Stacked local similarity trace and colormap of waveforms for two vibroseismic truck experiments. Denoted time on the title corresponds zero in time axis. (**b**) Detected nearby earthquake. (**c**) An unknown event, possibly associated with oil production. (**d**) An unknown event. The animations of these events can be found in the supplementary material (Animations S1–S4).

We apply the method to the Long Beach data between 6 March 2011 and 12 March 2011, covering the 11 March 2011 Mw 9.1 Tohoku-Oki sequence. We examine two frequency ranges, 5–10 Hz and <1 Hz, in order to detect local and distant events, respectively. The detection threshold is set as 10 MAD above the median. In the 5–10 Hz range, we detect 451 events (Table S1), whereas 401 are vibroseis truck signals from the petroleum survey. The vibroseis truck signals appear as sharp peaks in stacked local similarity with nearly every one-minute interval between 6 March and 9 March (Figs 6a, S1; Animation S1). In the remaining 50 events, three are nearby earthquakes listed in the SCEC catalog, one of which is a M 1.7 event from ~200 km away (Fig. 6b; Animation S2).

We also detect some weak but long-duration events up to a few minutes. One such event is very close to a possible production well identified from Google map (Figure S2; Personal communication with Philip Maechling). Although their waveform amplitudes were weak, we are able to observe the wavefield propagation in the animation and pinpoint the epicenter (Fig. 6c, Animation S3). However, this association could be by coincidence because there are many production wells in the study region. Apart from these events, many others are un-interpretable due to lack of additional information at this stage. Figure 6d shows an event with a sharp onset and a secondary phase and then followed by an emergent wavetrain. The animation suggests that both are from the same origin, but the underlying mechanism remains unclear (Animation S4).

We locate these 50 events with grid search over possible local source locations using a coarse grid size of 1 × 1 km. By shifting and stacking the local similarity traces, we take the source location corresponding the maximal stacked peak as the final location. Excluding the events with locations at the box boundaries (which are mostly unreliable), we obtained the locations of 35 events (Figure S3), none of them were listed in the SCSN catalog. The locations are scattered but have a weak NW-SE trend. Due to insufficient seismicity in this study, it is not clear if these events are associated with the local fault structure.

**Figure 7:**
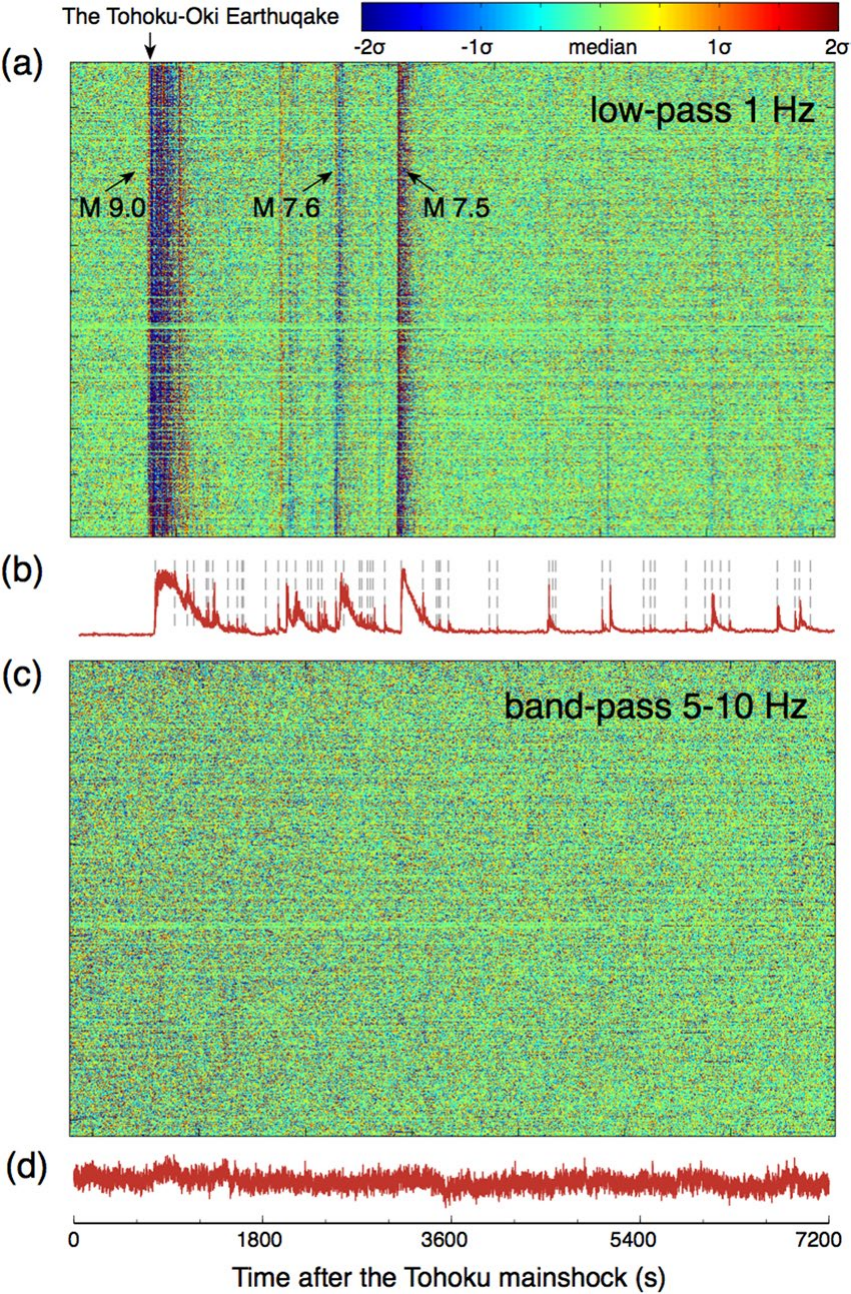
Waveforms and detection of two-hour Long Beach data after the Mw 9.1 Tohoku-Oki mainshock. (**a**) Lowpass 1 Hz waveforms recorded by the Long Beach array. (**b**) Stacked local similarity for lowpass 1 Hz data. The dashed gray lines mark the detected events. (**c**) Bandpass 5–10 Hz waveforms recorded by the Long Beach array. (**d**) Stacked local similarity for bandpass 5–10 Hz data. Note that no significant peaks are observed on the trace.

On the other hand, in the <1 Hz frequency range, we detect 183 distant events (Table S2). There were 125 events after the M 9.1 Tohoku earthquake and 101 matched with the earthquakes listed in the Japan Meteorological Agency (JMA) catalog. These earthquakes are far from Long Beach and, therefore, have very small amplitudes (Fig. 7a). The events not matching the JMA catalog could be possibly missing foreshocks or aftershocks^13^ or seismicity elsewhere around the world^23^. Figure 7 shows the detection of the aftershocks within two hours after the occurrence of the mainshock. From the waveform recordings, we observe three main events, corresponding to the M 9.1, M 7.6 and M 7.5 events in the JMA catalog and a few other weaker events (Fig. 7a). The stacked local similarity shows clear evidence of these and many other smaller events (Fig. 7b). Our sliding-window threshold method detects 56 events from the stacked local similarity, including those buried in the coda of large events.

Finally, we also examine the number of local detections (in the frequency range of 5–10 Hz) 6 hours before and after the Tohoku-Oki mainshock and find two and three events, respectively. However, none of them occurred during the passing surface waves of the mainshock. Hence, with our local similarity method, we are unable to observe any statistically significant change in local seismicity (Fig. 7c–d) associated with the Tohoku-Oki mainshock.

## Discussions

In this study, we introduced a new metric named local similarity based on waveform cross-correlations on spatially close stations for large-N arrays. By comparing a trace with those of neighboring stations, local similarity can effectively eliminate high-frequency spikes that likely contaminate conventional amplitude-based metrics (e.g., envelope or STA/LTA). This feature enables us to detect very weak events (up to 0.01 SNR in this case) with data recorded in noisy environments. We applied this method to one week of continuous data during the 2011 Mw9.1 Tohoku-Oki mainshock. While we detected many distant events in the frequency range of <1 Hz (most likely aftershocks), we did not observe any statistically significant change in events in the 5–10 Hz frequency range following the mainshock.

As shown in Fig. 6, local similarity can identify both impulsive and emergent events, which renders broad applicability, e.g., short-duration regular earthquakes and long-duration tectonic/volcanic tremor. In addition, direct stacking removes any wave type assumption or velocity model dependence, making it useful for detecting both local and distant events. Hence, our method could be used for detecting new types of events, as demonstrated by those interesting local events detected in the 5–10 Hz range (Fig. 6). However, direct stacking does not provide any locations or focal mechanisms. So additional methods are needed to further locate and classify event types. We note that when a certain class of events is targeted, a shift-stacking scheme can be applied to local similarity, such as used in the source-scanning algorithm^10^. With proper time shifts predicted by an assumed location and 1D velocity model, stacking of local similarity is expected to be more constructive. For example, to detect a local earthquake, we can apply theoretical travel time shifts of *P* (or *S*) waves to stack local similarity. This could result in a source-scanning-like or backprojection-like algorithm, but with local similarity traces rather than an envelope or normalized waveforms.

Figure 5 shows an interesting relationship between SNR and detection significance. Traditional seismology mainly focuses on events above the noise level, which fall in quadrant I (Fig. 5c). Some studies using template matching have achieved the capability to detect events below noise level^28^. However, it is unclear if events below the noise level are detectable without known templates. Using local similarity applied to ultra-dense arrays, we are able to detect events in a wide range below noise levels with high confidence (quadrant II in Fig. 5c).

Inbal *et al*.^4,5^ applied a backprojection method to 6-month recordings of the Long Beach array and detected many seismic events in the lower crust and upper mantle. To suppress local noise, they used downward continuation to back propagate the wavefield from the surface to 5 km depth. In comparison, in this study, we only ran on one-week data and used the original data without downward continuation. Currently, our method only performs grid search over horizontal space but not depth, and, hence, we do not have the accurate information on the depths of those detected events. However, by visually inspecting the animations (e.g., Animations S1–S4), we find most of them likely have shallow origins instead of from the lower crust or upper mantle. We have checked and found that none of our detections matched with Inbal *et al*.’s in the examined one-week window. Such difference could be mostly due to their use of downward continuation, which enhances detections of events at greater depths but suppresses detections of events less than 5 km.

Recent years have seen the exciting emergence of other dense nodal arrays like the Long Beach 3D array. Other examples include the San Jacinto Fault in California^29^, Sweetwater in Texas, Mount St Helens in Washington^30^, Piton de la Fournaise volcano in La Reunion^31^, and the 2016 IRIS community wavefield experiment. Our local similarity method could be potentially applied to these large-N datasets to detect weak and unknown seismic events. We note that the only requirement of this method is that the target signals on neighboring stations are more correlated than the background noise. Thus it does not require ultra-dense arrays with hundreds or thousands of stations. As long as the station spacing is comparable to or less than expected wavelengths, it can be applied to either 1D linear array or 2D array with only a few tens of stations. In fact, we have applied this method to the 1D Hi-CLIMB array across Himalaya and Southern Tibet, and the 2D PASO array (~60 stations) near Parkfield, and have detected many interesting local signals^32^. Hence, this method has the potential to be applied for event detections with traditional dense arrays as well.

## Electronic supplementary material

Animation S1

Animation S2

Animation S3

Animation S4

Supplementary information
