# Supplementary material for: High-resolution seismic event detection using local similarity for Large-N arrays
Source: Sci Rep. 2018 Jan 26;8:1646. doi: 10.1038/s41598-018-19728-w (PMC5786042; doi:10.1038/s41598-018-19728-w)
Supplement: Supplementary file 5 — Supplementary information [file 41598_2018_19728_MOESM5_ESM.pdf]

High-resolution seismic event detection using local similarity for Large-N arrays

Zefeng Li[1,2], Zhigang Peng[1], Dan Hollis[3], Lijun Zhu[4], and James McClellan[4]

1. School of Earth and Atmospheric Sciences, Georgia Institute of Technology, Atlanta, GA, USA

2. Seismological Laboratory, California Institute of Technology, Pasadena, CA, USA

3. Previously NodalSeismic Inc., currently Sisprobe SAS, San Gabriel, CA, USA

4. Center for Energy and Geo Processing at Georgia Tech and King Fahd University of Petroleum and Minerals

*Corresponding author (zefengli@gps.caltech.edu)

Supplementary Information:

The supplementary information included in this document:

Figure S1: Stacked local coherence for the hours with vibroseis truck experiments.

Figure S2: Production wells possibly related with the detected event in Figure 4c.

Figure S3: The event locations determined from a grid search method.

Figure S4: Baseline removal on stacked local similarity.

Table S1: Detected events on the Long Beach array at 5-10 Hz.

Table S2: Detected events on the Long Beach array at low-pass 1 Hz.

Legends of Animations S1-S4: The animations of the detected events shown in Figure 5a-5d. Animations S1-S4 (.mov files) can be downloaded online.

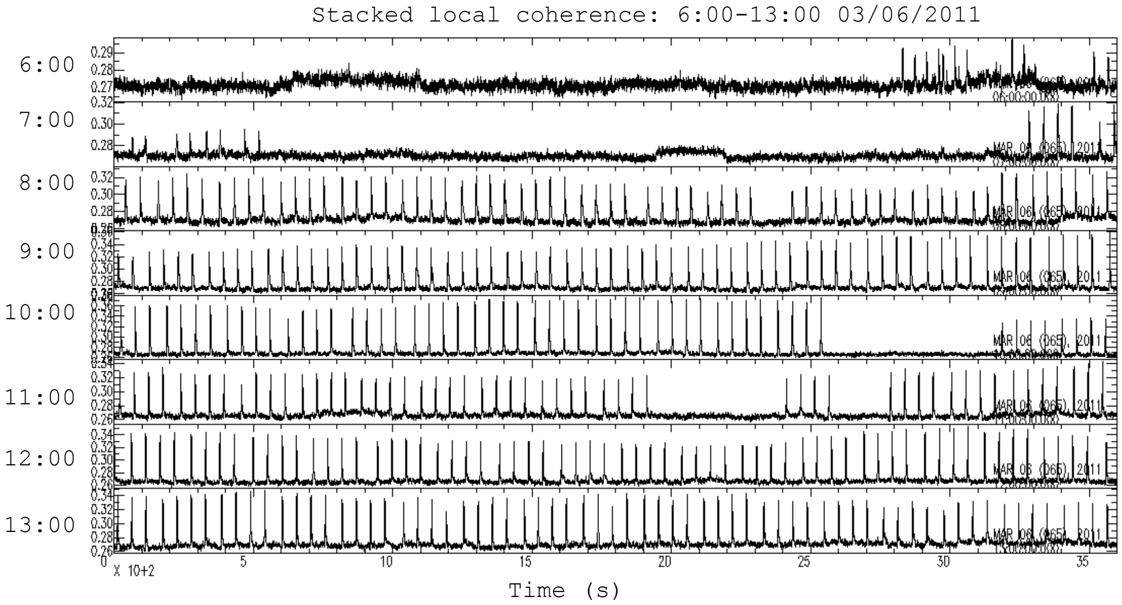

Figure S1. Stacked local coherence for the hours with vibroseis truck experiments.

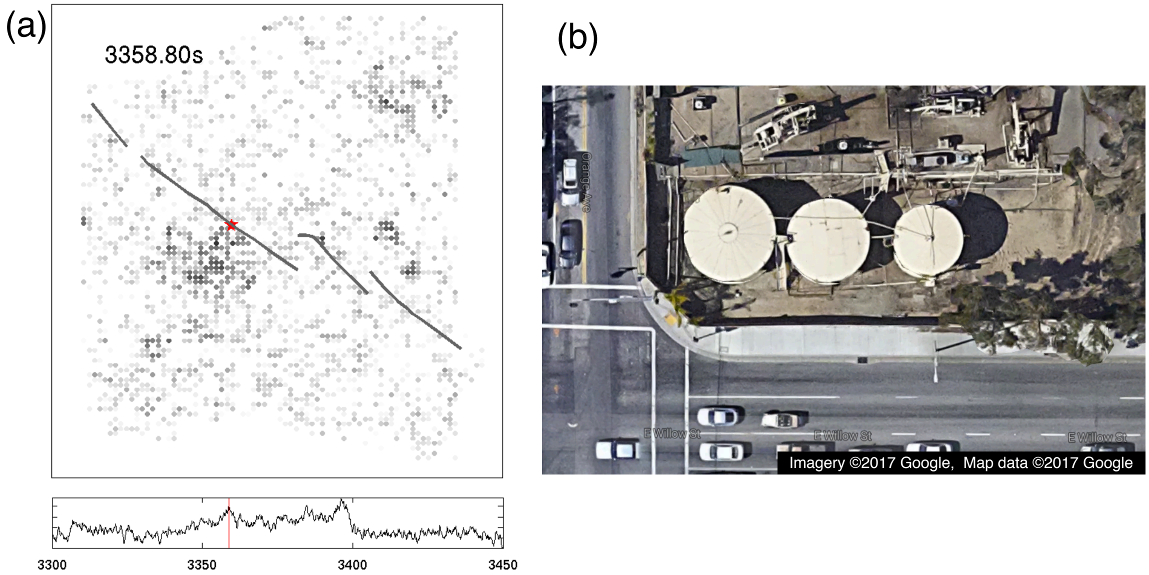

Figure S2. Spatial location of a detected event in Figure 4c and a production well. (a) A snapshot of the local coherence movie of the detected event. Red star mark the well location (33.80456, -118.17605) read in Google map. The gray line shows the Newport-Inglewood fault. Note the signal initiated near the well but then propagated southward. (b) A production well observed from Google map.

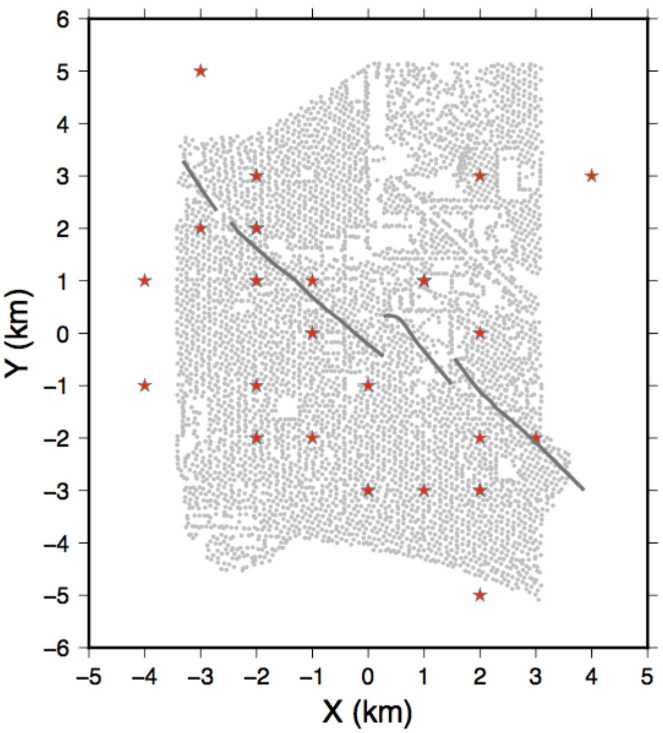

Figure S3. The event locations determined from a grid search method. Red stars are locations of detected events. Gray dots are the seismic sensors. Thick gray lines are the surface trace of the local Newport-Inglewood fault.

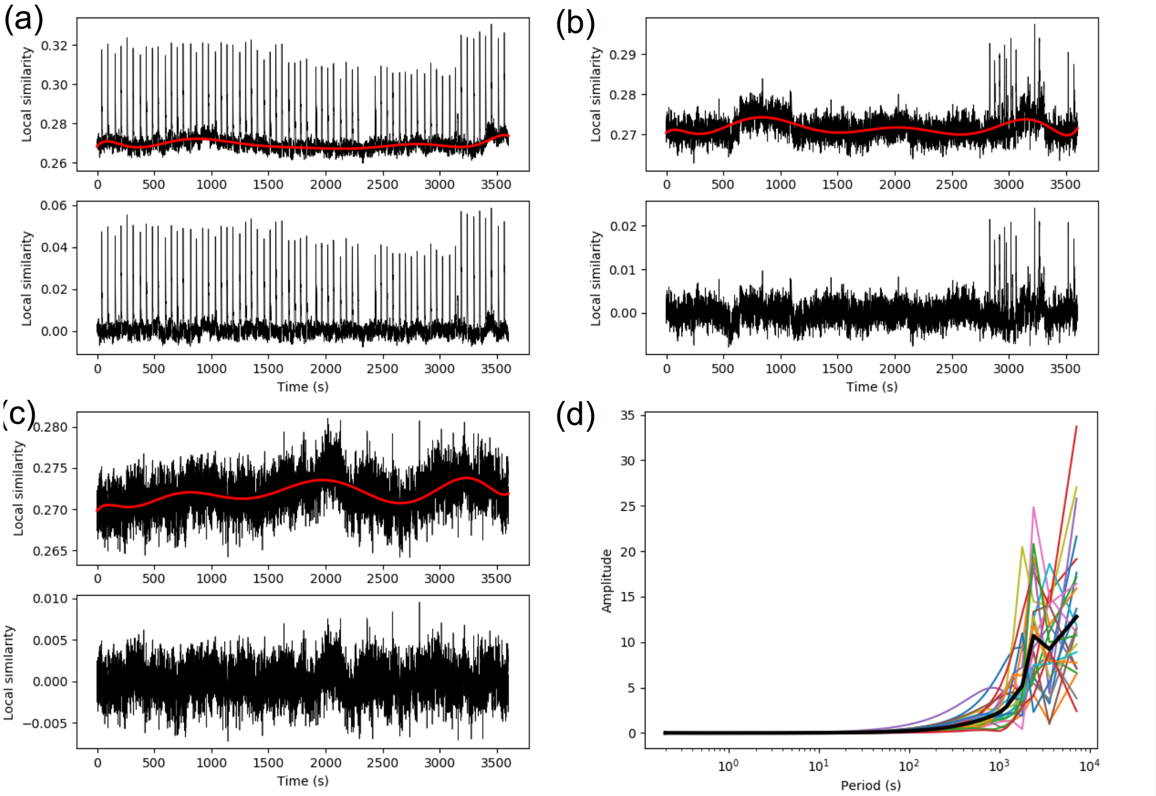

Figure S4. Baseline removal of stacked local similarity. (a)-(c) are the best fit 10th polynomials for examples with frequent peaks, scarce peaks, and no peaks. In the upper panel, the black lines are stacked local similarity and the red lines are the best-fitting polynomial baseline. The lower panel shows the local similarity with the baseline removed. (d) The spectra of the fit polynomials for 24 hourly local similarity traces on 6 March 2011. The main period fit by 10th polynomials concentrates on > 1000 s (~18 min).

Table S1: Detected events on the Long Beach array at 5-10 Hz

Year	month	day	hh:mm:ss	TimesofMAD	Year	month	day	hh:mm:ss	TimesofMAD
2011	3	6	6:47:10	18.2	2011	3	6	11:10:14	48
2011	3	6	6:47:54	12.5	2011	3	6	11:11:16	46.3
2011	3	6	6:48:37	13.4	2011	3	6	11:12:08	47
2011	3	6	6:49:19	13.3	2011	3	6	11:13:00	42.8
2011	3	6	6:49:35	11.2	2011	3	6	11:13:49	43.1
2011	3	6	6:50:17	14	2011	3	6	11:14:48	35.9
2011	3	6	6:51:01	11.9	2011	3	6	11:15:39	29.5
2011	3	6	6:53:43	16	2011	3	6	11:16:30	47.4
2011	3	6	6:54:26	22.8	2011	3	6	11:17:22	48.3
2011	3	6	6:54:44	10.8	2011	3	6	11:18:23	43.3
2011	3	6	6:55:05	12.3	2011	3	6	11:19:14	41.2
2011	3	6	6:58:37	15.6	2011	3	6	11:20:05	37.8
2011	3	6	6:59:28	10.6	2011	3	6	11:20:57	43.4
2011	3	6	7:01:06	15.6	2011	3	6	11:22:00	41.3
2011	3	6	7:01:53	16.7	2011	3	6	11:22:52	39
2011	3	6	7:03:44	17.2	2011	3	6	11:23:43	39.1
2011	3	6	7:04:32	18.3	2011	3	6	11:24:35	38.1
2011	3	6	7:05:32	19.1	2011	3	6	11:25:37	37.9
2011	3	6	7:06:20	18.5	2011	3	6	11:26:28	40.3
2011	3	6	7:07:48	18.8	2011	3	6	11:27:20	41
2011	3	6	7:08:40	17.3	2011	3	6	11:28:11	43.2
2011	3	6	7:35:52	11.6	2011	3	6	11:29:18	34.9
2011	3	6	7:54:44	28.2	2011	3	6	11:30:10	35.8
2011	3	6	7:55:36	37	2011	3	6	11:31:00	48.4
2011	3	6	7:56:27	39.2	2011	3	6	11:31:52	50.7
2011	3	6	7:57:17	38.5	2011	3	6	11:40:13	40.4
2011	3	6	7:58:57	25.6	2011	3	6	11:41:04	46.4
2011	3	6	7:59:49	24.7	2011	3	6	11:41:54	48.3
2011	3	6	8:00:41	41.2	2011	3	6	11:42:46	53.1
2011	3	6	8:01:33	43.2	2011	3	6	11:46:27	54.7
2011	3	6	8:02:38	39.5	2011	3	6	11:47:18	58.7

2011	3	6	8:03:30	35.3	2011	3	6	11:48:09	52.9
2011	3	6	8:04:22	39.6	2011	3	6	11:49:00	49.5
2011	3	6	8:05:15	36.1	2011	3	6	11:50:06	49.3
2011	3	6	8:06:18	33.3	2011	3	6	11:50:57	40.5
2011	3	6	8:07:11	36.1	2011	3	6	11:51:49	40.4
2011	3	6	8:08:03	35.1	2011	3	6	11:52:41	43.7
2011	3	6	8:08:54	35.8	2011	3	6	11:53:49	44.1
2011	3	6	8:09:58	31.6	2011	3	6	11:54:40	51.8
2011	3	6	8:10:50	39.5	2011	3	6	11:55:33	52.7
2011	3	6	8:11:41	37.3	2011	3	6	11:56:24	43.7
2011	3	6	8:12:33	32.9	2011	3	6	11:57:25	44.2
2011	3	6	8:13:39	32.6	2011	3	6	11:58:16	44
2011	3	6	8:14:30	31.5	2011	3	6	11:59:08	45.8
2011	3	6	8:15:23	36.8	2011	3	6	11:59:58	17.4
2011	3	6	8:16:15	36.5	2011	3	6	12:01:02	55.8
2011	3	6	8:17:15	34.9	2011	3	6	12:01:53	56.3
2011	3	6	8:18:07	38.7	2011	3	6	12:02:45	53
2011	3	6	8:18:57	38.1	2011	3	6	12:03:36	56.3
2011	3	6	8:19:48	34.9	2011	3	6	12:04:37	55.6
2011	3	6	8:20:48	31.4	2011	3	6	12:05:29	57.2
2011	3	6	8:21:40	38.2	2011	3	6	12:06:20	62.6
2011	3	6	8:22:29	39.4	2011	3	6	12:07:11	60.3
2011	3	6	8:23:21	33.3	2011	3	6	12:08:19	56.3
2011	3	6	8:24:22	31.2	2011	3	6	12:09:11	55.6
2011	3	6	8:25:13	37	2011	3	6	12:10:04	54.8
2011	3	6	8:26:05	40.4	2011	3	6	12:10:54	58.9
2011	3	6	8:26:57	40.8	2011	3	6	12:11:55	55.1
2011	3	6	8:27:58	28.1	2011	3	6	12:12:47	52.6
2011	3	6	8:28:49	28	2011	3	6	12:13:37	48.4
2011	3	6	8:29:41	30	2011	3	6	12:14:29	52.3
2011	3	6	8:30:33	31.5	2011	3	6	12:15:45	42.3
2011	3	6	8:31:55	32	2011	3	6	12:16:37	46.2
2011	3	6	8:32:46	32.4	2011	3	6	12:17:28	59.1

2011	3	6	8:33:39	29.9	2011	3	6	12:18:19	60.5
2011	3	6	8:34:29	37.3	2011	3	6	12:19:22	44.4
2011	3	6	8:35:30	30.6	2011	3	6	12:20:13	50.4
2011	3	6	8:36:20	29.5	2011	3	6	12:21:04	62.1
2011	3	6	8:37:13	38.1	2011	3	6	12:21:56	61.8
2011	3	6	8:38:03	40.3	2011	3	6	12:23:04	58.7
2011	3	6	8:40:33	29.6	2011	3	6	12:23:55	46.1
2011	3	6	8:41:25	31.6	2011	3	6	12:24:48	46.7
2011	3	6	8:42:16	28.3	2011	3	6	12:25:40	48.1
2011	3	6	8:43:08	29.6	2011	3	6	12:26:46	43.9
2011	3	6	8:44:06	29.5	2011	3	6	12:27:37	42.1
2011	3	6	8:44:58	28	2011	3	6	12:28:28	45.4
2011	3	6	8:45:49	31.3	2011	3	6	12:29:19	44
2011	3	6	8:46:42	33.5	2011	3	6	12:30:22	44.1
2011	3	6	8:47:47	26.9	2011	3	6	12:31:13	43.6
2011	3	6	8:48:39	26.9	2011	3	6	12:32:05	43.8
2011	3	6	8:49:31	28.7	2011	3	6	12:32:56	40.6
2011	3	6	8:50:22	26.4	2011	3	6	12:33:58	36.5
2011	3	6	8:51:25	26.3	2011	3	6	12:34:49	44.2
2011	3	6	8:52:15	25.5	2011	3	6	12:35:41	44.1
2011	3	6	8:52:36	11.5	2011	3	6	12:36:32	44.5
2011	3	6	8:53:06	42.5	2011	3	6	12:37:35	42.4
2011	3	6	8:53:58	41.5	2011	3	6	12:38:27	42.5
2011	3	6	8:54:57	41.8	2011	3	6	12:39:17	52.1
2011	3	6	8:55:48	43.8	2011	3	6	12:40:09	53.7
2011	3	6	8:56:39	34.3	2011	3	6	12:41:12	61.1
2011	3	6	8:57:30	40.1	2011	3	6	12:42:02	61
2011	3	6	8:58:31	34.2	2011	3	6	12:42:54	54.6
2011	3	6	8:59:23	31.6	2011	3	6	12:43:46	50.8
2011	3	6	9:00:15	39.6	2011	3	6	12:44:51	56.8
2011	3	6	9:01:07	36.4	2011	3	6	12:45:41	63.4
2011	3	6	9:02:06	41.1	2011	3	6	12:46:33	70.2
2011	3	6	9:02:57	38.1	2011	3	6	12:47:23	72.9

2011	3	6	9:03:50	42	2011	3	6	12:48:30	64.9
2011	3	6	9:04:41	40.5	2011	3	6	12:49:23	59.5
2011	3	6	9:05:41	44.8	2011	3	6	12:50:13	66.4
2011	3	6	9:06:32	38	2011	3	6	12:51:04	69.3
2011	3	6	9:07:23	40.7	2011	3	6	12:52:04	61.6
2011	3	6	9:08:14	44.2	2011	3	6	12:52:57	61
2011	3	6	9:09:14	47.5	2011	3	6	12:53:47	54.6
2011	3	6	9:10:05	50.5	2011	3	6	12:54:38	63.4
2011	3	6	9:10:57	54.4	2011	3	6	12:55:38	52.8
2011	3	6	9:11:49	47.7	2011	3	6	12:56:28	50.5
2011	3	6	9:12:46	48.8	2011	3	6	12:57:20	59.3
2011	3	6	9:13:38	50.1	2011	3	6	12:58:13	58.2
2011	3	6	9:14:30	45.8	2011	3	6	12:59:21	47.9
2011	3	6	9:15:22	55.8	2011	3	6	13:00:13	45.2
2011	3	6	9:16:21	52.9	2011	3	6	13:01:04	45
2011	3	6	9:17:13	47.2	2011	3	6	13:01:55	47.4
2011	3	6	9:18:05	46.7	2011	3	6	13:02:55	55.8
2011	3	6	9:18:57	46.5	2011	3	6	13:03:45	59
2011	3	6	9:19:58	42.7	2011	3	6	13:04:36	56.3
2011	3	6	9:20:50	42.1	2011	3	6	13:05:27	47.1
2011	3	6	9:21:41	40.9	2011	3	6	13:06:26	52.2
2011	3	6	9:22:32	44.3	2011	3	6	13:07:17	54.1
2011	3	6	9:23:32	38.4	2011	3	6	13:08:10	58.5
2011	3	6	9:24:22	49.8	2011	3	6	13:09:01	58.9
2011	3	6	9:25:14	49.2	2011	3	6	13:10:04	50.5
2011	3	6	9:26:05	55.5	2011	3	6	13:10:56	60.1
2011	3	6	9:27:06	44.6	2011	3	6	13:11:47	58.2
2011	3	6	9:27:59	57.5	2011	3	6	13:12:39	43.3
2011	3	6	9:28:50	56.9	2011	3	6	13:13:39	39.8
2011	3	6	9:29:42	54.3	2011	3	6	13:14:30	49
2011	3	6	9:30:43	52.4	2011	3	6	13:15:20	46.6
2011	3	6	9:31:35	45.9	2011	3	6	13:16:11	47.8
2011	3	6	9:32:26	45.2	2011	3	6	13:17:19	46.3

2011	3	6	9:33:18	44.9	2011	3	6	13:18:09	52.1
2011	3	6	9:34:20	52.3	2011	3	6	13:19:00	43.7
2011	3	6	9:35:11	47.4	2011	3	6	13:19:52	31.7
2011	3	6	9:36:03	51.5	2011	3	6	13:20:55	46.8
2011	3	6	9:36:54	50.8	2011	3	6	13:21:47	50.1
2011	3	6	9:37:52	56	2011	3	6	13:22:38	54.1
2011	3	6	9:38:43	52.8	2011	3	6	13:23:29	58.7
2011	3	6	9:39:34	53.6	2011	3	6	13:24:33	56.8
2011	3	6	9:40:26	44.4	2011	3	6	13:25:22	58.1
2011	3	6	9:41:28	58	2011	3	6	13:26:12	56.5
2011	3	6	9:42:19	58.4	2011	3	6	13:27:03	46
2011	3	6	9:43:11	57.4	2011	3	6	13:28:05	53.8
2011	3	6	9:44:02	54.5	2011	3	6	13:28:57	54.7
2011	3	6	9:45:04	51.4	2011	3	6	13:29:48	39.2
2011	3	6	9:45:57	49.1	2011	3	6	13:30:40	47
2011	3	6	9:46:48	44.7	2011	3	6	13:31:43	47.1
2011	3	6	9:47:39	46.9	2011	3	6	13:32:34	46.4
2011	3	6	9:48:39	53.2	2011	3	6	13:33:25	45.7
2011	3	6	9:49:30	57.8	2011	3	6	13:34:17	51.6
2011	3	6	9:50:22	55.3	2011	3	6	13:35:17	47.8
2011	3	6	9:51:13	58.6	2011	3	6	13:36:08	46.1
2011	3	6	9:52:14	59	2011	3	6	13:36:58	51.3
2011	3	6	9:53:05	58.5	2011	3	6	13:37:50	51.9
2011	3	6	9:53:58	51.9	2011	3	6	13:38:52	48.2
2011	3	6	9:54:48	50.1	2011	3	6	13:39:44	50.3
2011	3	6	9:55:55	50.8	2011	3	6	13:40:36	42.8
2011	3	6	9:56:46	46.2	2011	3	6	13:41:28	46.6
2011	3	6	9:57:38	58.8	2011	3	6	13:42:28	47.3
2011	3	6	9:58:30	60.3	2011	3	6	13:43:20	40
2011	3	6	9:59:34	62.6	2011	3	6	13:44:11	42.4
2011	3	6	10:00:24	57.3	2011	3	6	13:45:02	42.3
2011	3	6	10:01:16	57.2	2011	3	6	13:46:02	35.2
2011	3	6	10:02:08	64.2	2011	3	6	13:46:53	35.7

2011	3	6	10:03:10	64.7	2011	3	6	13:47:46	41.2
2011	3	6	10:04:00	68.1	2011	3	6	13:48:37	38
2011	3	6	10:04:53	68.1	2011	3	6	13:49:38	34.9
2011	3	6	10:05:46	65.4	2011	3	6	13:50:30	35.7
2011	3	6	10:06:47	64.7	2011	3	6	13:51:22	43.4
2011	3	6	10:07:39	62.4	2011	3	6	13:52:14	35.4
2011	3	6	10:08:29	65.5	2011	3	6	13:53:16	35
2011	3	6	10:09:19	73.7	2011	3	6	13:54:07	38.7
2011	3	6	10:10:25	57.2	2011	3	6	13:54:56	37.5
2011	3	6	10:11:17	73.3	2011	3	6	13:55:48	33.4
2011	3	6	10:12:07	75.2	2011	3	6	13:56:50	36.5
2011	3	6	10:12:59	65.2	2011	3	6	13:57:41	42.6
2011	3	6	10:14:16	66.9	2011	3	6	13:58:32	42.4
2011	3	6	10:15:07	58.6	2011	3	6	13:59:24	38.5
2011	3	6	10:15:59	73.1	2011	3	7	1:07:05	19.1
2011	3	6	10:16:50	75.1	2011	3	7	6:18:25	10.1
2011	3	6	10:17:59	76.7	2011	3	7	8:23:52	12.8
2011	3	6	10:18:50	75.7	2011	3	7	8:33:55	49.7
2011	3	6	10:19:41	78.2	2011	3	7	9:35:48	10.8
2011	3	6	10:20:33	78.8	2011	3	7	11:11:26	10.6
2011	3	6	10:21:35	72.8	2011	3	7	11:32:15	10
2011	3	6	10:22:26	63.7	2011	3	7	14:15:23	10
2011	3	6	10:23:17	84.7	2011	3	7	16:17:05	10.2
2011	3	6	10:24:08	85.2	2011	3	7	21:57:35	10.1
2011	3	6	10:25:11	86.1	2011	3	8	5:45:05	11.2
2011	3	6	10:26:02	76.4	2011	3	8	9:25:34	11.2
2011	3	6	10:26:54	84.1	2011	3	8	9:26:09	12
2011	3	6	10:27:46	84.6	2011	3	8	9:55:47	10.7
2011	3	6	10:28:51	84	2011	3	8	10:04:46	14.3
2011	3	6	10:29:42	77.4	2011	3	8	10:14:21	11.3
2011	3	6	10:30:35	85.5	2011	3	8	10:17:40	10
2011	3	6	10:31:27	86	2011	3	8	10:45:22	15
2011	3	6	10:32:31	78.3	2011	3	8	10:49:49	12.2

2011	3	6	10:33:23	72.1	2011	3	8	11:03:28	15.3
2011	3	6	10:34:14	82.7	2011	3	8	11:09:48	10.8
2011	3	6	10:35:04	84.3	2011	3	8	11:16:33	35.1
2011	3	6	10:36:06	70.2	2011	3	8	11:24:24	10.4
2011	3	6	10:36:57	75.1	2011	3	8	12:11:16	10.3
2011	3	6	10:37:49	77.3	2011	3	8	12:14:38	10.7
2011	3	6	10:38:40	85.7	2011	3	8	17:08:43	11.6
2011	3	6	10:39:42	83.8	2011	3	9	3:09:04	10.7
2011	3	6	10:40:34	80.4	2011	3	9	4:17:55	58
2011	3	6	10:41:25	77.7	2011	3	9	11:21:18	12.1
2011	3	6	10:42:17	123.7	2011	3	9	11:32:07	11.1
2011	3	6	10:53:07	49.9	2011	3	9	12:17:42	15
2011	3	6	10:53:59	47.6	2011	3	10	5:05:30	10.9
2011	3	6	10:54:50	51.2	2011	3	10	6:12:09	10.3
2011	3	6	10:55:41	51.9	2011	3	10	11:02:08	11.9
2011	3	6	10:56:42	49.7	2011	3	11	3:22:16	10.7
2011	3	6	10:57:34	50.8	2011	3	11	4:51:55	51.5
2011	3	6	10:58:26	58.9	2011	3	11	9:34:59	11.6
2011	3	6	10:59:19	57.5	2011	3	11	10:15:47	11.7
2011	3	6	11:00:20	52.4	2011	3	11	11:16:22	10.2
2011	3	6	11:01:12	52.1	2011	3	11	13:55:42	13.3
2011	3	6	11:02:04	45	2011	3	11	14:39:00	31.4
2011	3	6	11:02:55	46.6	2011	3	12	6:31:07	11.3
2011	3	6	11:04:00	41	2011	3	12	6:31:33	12.5
2011	3	6	11:04:51	53.1	2011	3	12	7:47:49	10.7
2011	3	6	11:05:43	53.5	2011	3	12	8:55:58	11.3
2011	3	6	11:06:34	50.9	2011	3	12	8:56:36	15.1
2011	3	6	11:07:37	40.6	2011	3	12	11:30:43	11
2011	3	6	11:08:30	55.3	2011	3	12	12:53:59	10.7
2011	3	6	11:09:22	41.8					

Table S2: Detected events on the Long Beach array at low-pass 1 Hz

Year	month	day	hh:mm:ss	TimesofMAD	Year	month	day	hh:mm:ss	TimesofMAD
2011	3	6	2:57:37	10.4	2011	3	11	6:52:47	13.5
2011	3	6	4:24:31	10.5	2011	3	11	7:00:59	70.9
2011	3	6	11:20:49	10.4	2011	3	11	7:01:35	19.1
2011	3	6	12:42:57	120.3	2011	3	11	7:02:00	10.2
2011	3	6	14:51:15	36	2011	3	11	7:09:26	52.7
2011	3	6	14:52:45	31	2011	3	11	7:10:44	78.6
2011	3	7	0:22:42	23.3	2011	3	11	7:16:02	10.8
2011	3	7	1:11:46	11.4	2011	3	11	7:17:06	14.8
2011	3	7	1:26:17	10	2011	3	11	7:17:46	11
2011	3	7	6:52:28	13.6	2011	3	11	7:22:47	27.3
2011	3	7	19:22:32	17.6	2011	3	11	7:26:00	13
2011	3	7	22:38:19	12.9	2011	3	11	7:26:57	53.4
2011	3	8	10:09:17	11.1	2011	3	11	7:28:18	10.5
2011	3	8	14:23:35	10.1	2011	3	11	7:29:47	30.2
2011	3	8	18:35:55	10	2011	3	11	7:37:17	47
2011	3	8	21:15:01	10.1	2011	3	11	7:40:07	57.3
2011	3	8	21:44:02	10.2	2011	3	11	7:40:57	61.8
2011	3	9	2:57:29	35.5	2011	3	11	7:42:43	13.2
2011	3	9	2:57:55	12.4	2011	3	11	7:46:43	11.2
2011	3	9	3:33:23	10.9	2011	3	11	7:47:52	24.4
2011	3	9	4:18:03	24.1	2011	3	11	7:55:53	12.6
2011	3	9	4:26:37	10	2011	3	11	7:56:35	16.1
2011	3	9	4:31:12	14	2011	3	11	8:06:35	18.2
2011	3	9	4:48:58	23	2011	3	11	8:13:55	19.4
2011	3	9	5:30:49	10	2011	3	11	8:16:46	20.6
2011	3	9	7:54:14	10.1	2011	3	11	8:18:15	10.5
2011	3	9	9:37:02	12.8	2011	3	11	8:24:03	73.8
2011	3	9	9:37:42	10	2011	3	11	8:27:24	74
2011	3	9	10:06:40	11.4	2011	3	11	8:31:36	99.1
2011	3	9	13:31:17	13.7	2011	3	11	8:38:36	11.4
2011	3	9	18:28:06	10.1	2011	3	11	8:39:39	38.7

2011	3	9	18:56:42	12.9	2011	3	11	8:43:13	45.4
2011	3	9	21:36:04	23.4	2011	3	11	8:44:44	32.1
2011	3	9	23:15:17	12.1	2011	3	11	8:53:01	45.8
2011	3	9	23:48:52	12	2011	3	11	9:04:29	12.4
2011	3	10	1:34:43	11	2011	3	11	9:34:01	13.1
2011	3	10	1:58:12	13.3	2011	3	11	9:50:57	10.2
2011	3	10	2:11:47	10.3	2011	3	11	9:51:49	10.7
2011	3	10	2:20:34	10.3	2011	3	11	10:22:36	22.3
2011	3	10	4:18:13	10.1	2011	3	11	10:23:03	10.8
2011	3	10	7:54:55	10.3	2011	3	11	10:23:52	11.8
2011	3	10	8:09:34	12	2011	3	11	10:32:30	12.7
2011	3	10	8:11:57	10.7	2011	3	11	10:40:24	49.4
2011	3	10	8:20:07	10.7	2011	3	11	10:42:12	10.6
2011	3	10	8:20:37	11.9	2011	3	11	10:57:27	15.2
2011	3	10	10:38:14	10.7	2011	3	11	11:12:44	23.2
2011	3	10	10:47:56	10	2011	3	11	11:12:59	10.8
2011	3	10	10:54:45	14.5	2011	3	11	11:25:10	16.7
2011	3	10	11:24:27	10.5	2011	3	11	11:28:46	14.8
2011	3	10	12:11:57	10.9	2011	3	11	11:33:16	24.3
2011	3	10	14:35:26	10.8	2011	3	11	11:44:08	36
2011	3	10	14:45:25	10.1	2011	3	11	11:48:39	86.9
2011	3	10	15:57:15	10.7	2011	3	11	11:51:35	19.4
2011	3	10	17:26:39	129.8	2011	3	11	11:56:24	15.6
2011	3	10	17:28:22	17.9	2011	3	11	12:24:57	35.4
2011	3	10	17:28:42	17	2011	3	11	13:01:09	17.8
2011	3	11	4:29:05	10.9	2011	3	11	13:28:58	29
2011	3	11	4:51:55	98.9	2011	3	11	13:54:50	21.1
2011	3	11	5:58:27	18.3	2011	3	11	15:08:15	24.3
2011	3	11	6:01:33	10.6	2011	3	11	15:26:05	24.5
2011	3	11	6:03:31	17.5	2011	3	11	15:32:13	26.6
2011	3	11	6:04:31	16	2011	3	11	15:33:18	11.2
2011	3	11	6:06:33	10.3	2011	3	11	18:23:19	22
2011	3	11	6:06:50	14	2011	3	11	18:29:03	39.6

2011	3	11	6:07:49	41.3	2011	3	11	19:06:38	10.4
2011	3	11	6:10:07	12.1	2011	3	11	19:11:30	55.7
2011	3	11	6:11:28	19.1	2011	3	11	19:14:43	48.7
2011	3	11	6:12:11	10.9	2011	3	11	19:58:54	57.8
2011	3	11	6:12:45	28.8	2011	3	11	20:23:17	15.6
2011	3	11	6:15:57	25.2	2011	3	12	1:30:43	20.2
2011	3	11	6:17:57	29.5	2011	3	12	1:59:16	29.2
2011	3	11	6:19:16	42.6	2011	3	12	3:13:32	21.4
2011	3	11	6:21:48	25	2011	3	12	3:23:58	23.5
2011	3	11	6:22:40	10	2011	3	12	4:43:52	13.1
2011	3	11	6:23:14	10.9	2011	3	12	5:23:40	10.3
2011	3	11	6:24:21	27.2	2011	3	12	7:14:33	10
2011	3	11	6:25:19	30.7	2011	3	12	8:07:22	11.1
2011	3	11	6:27:05	23.1	2011	3	12	10:34:39	10
2011	3	11	6:29:03	18.1	2011	3	12	10:46:31	14.9
2011	3	11	6:31:02	12.4	2011	3	12	11:05:17	35.1
2011	3	11	6:31:13	14.5	2011	3	12	12:00:32	10.1
2011	3	11	6:32:09	11.1	2011	3	12	12:26:30	11.2
2011	3	11	6:32:41	16.7	2011	3	12	12:38:58	12.2
2011	3	11	6:33:16	38.9	2011	3	12	12:39:25	13.1
2011	3	11	6:34:52	55.4	2011	3	12	13:05:54	25.9
2011	3	11	6:37:33	100.3	2011	3	12	13:27:50	37.7
2011	3	11	6:41:00	37.9	2011	3	12	13:28:57	11.7
2011	3	11	6:43:07	11.2	2011	3	12	14:13:49	41.5
2011	3	11	6:43:27	13.8	2011	3	12	14:15:26	15.6
2011	3	11	6:43:39	21.1	2011	3	12	14:55:03	13
2011	3	11	6:45:03	28.6	2011	3	12	15:14:31	15.4
2011	3	11	6:51:34	10.9					

Animation S1: Wave propagation of a detected seismic event 2011030609. Upper panel: The dots represent sensor locations, gray-coded by the values of local coherence at the time step. Darker dots represent higher local coherence values, and vice versa. The black curves mark the surface trace of the Newport-Inglewood fault. Time stamp at the left top is relative to the beginning of that hour. Lower panel: the stacked local coherence. The red bar marks the time step.

Animation S2: Wave propagation of a detected seismic event 2011030708. Symbols are the same as those in Animation S1.

Animation S3: Wave propagation of a detected seismic event 2011031208. Symbols are the same as those in Animation S1. The red star marks the production wells like facility observed from Google map.

Animation S4: Wave propagation of a detected seismic event 2011030811. Symbols are the same as those in Animation S1.
